# The Influence of Communication Sample Length on Reliability and Convergent Validity of Vocal Measures Derived From the Communication Complexity Scale

**DOI:** 10.1044/2022_JSLHR-22-00010

**Published:** 2022-09-12

**Authors:** Jena McDaniel, Nancy C. Brady

**Affiliations:** a Life Span Institute, The University of Kansas, Lawrence; b Department of Speech-Language-Hearing: Sciences & Disorders, The University of Kansas, Lawrence

## Abstract

**Purpose:**

This study examines the effects of communication sample length on the reliability and convergent validity of six vocal measures for children with autism spectrum disorder (ASD) with minimal verbal skills. The results are expected to inform recommendations for the length of communication samples for clinical and research purposes.

**Method:**

Participants included 31 children with ASD (24 boys and seven girls; *M*_age_ = 6;7 [years;months], *SD* = 17 months) with minimal verbal skills. We coded six vocal measures that focus on vocalizations and early word productions from the Communication Complexity Scale (CCS) scripted administration protocol. To evaluate reliability of different sample lengths, we calculated intraclass correlation coefficients between the full CCS sample and 1-, 3-, 7-, 10-, and 20-min samples. To examine convergent validity, we calculated correlations between the six vocal measures for each sample length.

**Results:**

When coded from 10-min samples from the beginning of the CCS, all of the vocal measures exhibit adequate reliability with the full CCS sample. Some vocal measures exhibit adequate reliability with samples as short as 3 min. For convergent validity, all of the correlations between the vocal measures exceed .40 and are statistically significant for the 10-min samples except for some of the correlations with the proportion of communicative vocalizations. Similar results were found for other sample lengths.

**Conclusion:**

Findings support coding 10-min segments from the CCS scripted administration protocol to evaluate the vocal development skills of children with ASD with minimal verbal skills.

**Supplemental Material:**

https://doi.org/10.23641/asha.20999938

The necessary length of a communication sample is an important question for speech-language pathologists (SLPs) and researchers. Both groups of professionals need samples of sufficient length for stable results while avoiding longer-than-necessary samples that waste time and resources. SLPs report lack of time as one of the primary reasons for not conducting language samples (Kemp & Klee, 1997; Pavelko et al., 2016). If relatively shorter samples can provide results similar to those from longer samples, SLPs may be able to use them more often and gain rich, essential knowledge about an individual's communication skills.

The impact of sample length on the stability and reliability of word-level measures, such as mean length of utterance (MLU), has been investigated to a limited degree. Heilmann et al. (2010) reported that 3-min samples exhibited sufficient reliability (Cronbach's alpha ≥ .80) with 7-min samples for number of total utterances, words per minute, and number of different words for preschool children with typical language. The results for MLU in morphemes were somewhat less strong than the other measures. Guo and Eisenberg (2015) reported that total number of words reached sufficient reliability (*r* ≥ .90) with 7-min samples for 6-year-old children with typical language, but number of different words and mean length of C-units in morphemes required 10-min samples to reach this criterion.

However, few investigations have focused on vocal measures, which are especially important for children using presymbolic and early symbolic communication. For children who are not yet talking, such as children with autism spectrum disorder (ASD) with minimal verbal skills, SLPs need measures to evaluate progress for intervention planning. Measures of prelinguistic vocal skills are strong candidates given the continuity between prelinguistic vocalizations and spoken words (McCune & Vihman, 2001; Oller, 2000; Rvachew et al., 2006; Stoel-Gammon, 1991; Vihman, 2017; Vihman et al., 1985; Watt et al., 2006). This continuity aligns with multiple theories of language development (e.g., social feedback theory [Goldstein et al., 2003; Goldstein & Schwade, 2008], social feedback loop theory [Warlaumont et al., 2014], and transactional theory of spoken language development [Camarata & Yoder, 2002; McLean & Snyder-McLean, 1978]).

A number of vocal measures have been evaluated as predictors and/or outcome measures for children with ASD. For the current analyses, we focus on six variables relevant to children with ASD with minimal verbal skills: total vocalizations, communicative vocalizations, proportion of communicative vocalizations, consonant inventory, diversity of key consonants used in communication acts (DKCC), and word approximations. There is empirical support for the use of each of these measures in describing a child's vocal productions. Prior work provides empirical and theoretical support for considering these vocal measures for use in assessing and predicting progress (e.g., McDaniel, 2019; McDaniel et al., 2018, 2020b; Wetherby et al., 2007; Yoder et al., 2015). McDaniel et al. (2020a, 2020b) presented evidence of convergent validity, divergent validity, and sensitivity to change for the number of communicative vocalizations, proportion of communicative vocalizations, and DKCC. Wetherby et al. (2007), Woynaroski et al. (2014, 2016, 2017), and Yoder et al. (2015) offer additional support for the convergent validity and sensitivity to change for DKCC. The proportion of communicative vocalizations and DKCC were also shown to be sufficiently stable (intraclass correlation coefficient [ICC] ≥ .80) with a single 6-min communication sample from the Early Communication Indicator (Greenwood et al., 2011, 2020; McDaniel et al., 2021; Priest et al., 2001). Plumb (2008) provides additional support for the convergent validity of communicative vocalizations. For total vocalizations, Brian et al. (2017), McDaniel (2019), and Plumb (2008) provide evidence for convergent validity and sensitivity to change. In addition to DKCC, we included consonant inventory as an indication of the different speech sounds a child produces in any context (Paul et al., 2008). Finally, we included word approximations because they serve as a bridge to spoken word productions and are often reported in measures such as number of different words produced.

As a next step in translating psychometric evidence for the different vocal measures to clinical practice, we investigate how sample length influences the reliability and convergent validity of the vocal variables. If shorter samples exhibit sufficient psychometric properties, it would be more feasible to use the vocal measures in clinical practice and for research than if longer samples are needed.

## Purpose and Research Question

The current study was designed to examine the effects of communication sample length on the reliability and convergent validity of six vocal measures for children with ASD who have minimal verbal skills. The current analysis extends prior work by evaluating the influence of different sample lengths. The results will provide guidance for the necessary length of communication samples for deriving representative measures of early vocal behavior. We addressed two research questions.

1. Do vocal measures from communication samples exhibit adequate reliability when comparing longer versus shorter segments as determined by Cronbach's alpha values?
2. Do vocal measures from communication samples of varying lengths exhibit evidence of convergent validity as determined by correlations between the vocal measures?

## Method

Caregivers provided written informed consent before participants began the study. The University of Kansas Institutional Review Board approved all study procedures.

### Participants

Participants were drawn from a larger intervention study evaluating the effect of a multicomponent intervention on the production of target words for school-age children with ASD and minimal verbal skills (Brady et al., 2021). For study recruitment, special education teachers and SLPs distributed flyers and consent forms within approved school districts. Measures for the current analysis were collected prior to the start of the intervention to eliminate the intervention's influence on the findings. The 31 participants (24 boys and seven girls) had a mean age of 6;7 (years;months; *SD* = 17 months, range: 4;7–10;10) and either used no spoken words or only used single words. Participant characteristics prior to study entry are shown in Table 1. The Autism Diagnostic Observation Schedule (Lord et al., 2012) comparison score is presented as a measure of ASD symptom severity. A comparison score of 1 indicates minimal to no evidence of ASD-related symptoms. A score of 10 indicates high impairment. Six participants were reported to be Hispanic, and 25 were reported to be non-Hispanic. Thirteen children were reported to be White, 10 were reported to be more than one race, five were reported to be Black or African American, and three were reported to be Asian. Socioeconomic status data were not collected.

**Table 1.** Participant characteristics at study entry.

Measure	*M*	*SD*	Min	Max
Chronological age (months)	79	17	55	130
Number of words understood on MCDI (raw score)	186	172	7	394
Number of words said on MCDI (raw score)	58	100	0	362
MSEL Visual Reception (raw score)	24.45	8.45	3	41
MSEL Visual Reception (*t* score)	19.03	0.19	19	20
VABS Adaptive Skills (raw score)	220.24	47.67	143	316
VABS Adaptive Skills (standard score)	55.32	9.31	40	74
VABS Communication (raw score)	13.76	6.94	5	30
VABS Communication (standard score)	45.24	13.97	24	74
VABS Daily Living Skills (raw score)	20.44	6.63	8	38
VABS Daily Living Skills (standard score)	58.40	10.74	37	85
VABS Socialization (raw score)	18.72	7.40	6	33
VABS Socialization (standard score)	50.04	16.54	21	79
VABS Motor Skills (raw score)	17.60	6.36	2	29
VABS Motor Skills (standard score)	66.56	19.46	1	95
ADOS comparison score	7.59	1.10	6	10

*Note.* Min = minimum; Max = maximum; MCDI = MacArthur–Bates Communicative Development Inventory (Fenson et al., 2007); MSEL = Mullen Scales of Early Learning (Mullen, 1995); VABS = Vineland Adaptive Behavior Scales (Sparrow et al., 2005); ADOS = Autism Diagnostic Observation Schedule (Lord et al., 2012).

### Procedure

For this study, we coded six measures that focus on vocalizations and early word productions as shown in Table 2. The sampling context was the Communication Complexity Scale (CCS) scripted administration protocol (Brady et al., 2018). The CCS is designed to evaluate expressive communication skills of individuals who primarily communicate with presymbolic or early symbolic means during 12 play-based activities. The activities are presented in the following order: wind-up toy, blocks, snack, musical instruments, hammer toy with sound effects, fan that lights up, magnetic tiles, dot paint, bubbles, books, bumble toys that vibrate, and ball popper toy. The activities alternate between ones designed to elicit requests in particular and those designed to elicit comments. For example, a broken wind-up toy is used in the first activity to elicit requests. The block activity includes a surprising pretend bug designed to elicit comments. Prior to the wind-up toy activity, the examiner provides a brief warm-up activity designed to build rapport with the participant. The participant is permitted to play with one of three toys with no task demands for approximately 5 min, depending on the child's apparent enjoyment of the activity. Examiners were trained to administer the CCS reliably. For the most part, they were unfamiliar with the participants prior to administration. Although the CCS scripted administration protocol has its own scoring system for pre-intentional, intentional symbolic, and beginning symbolic communication, it does not quantify or describe vocalizations in detail. Coding vocalizations from the already collected CCS presents an opportunity to examine vocal productions during observed communication interactions.

**Table 2.** Operational definitions of coded measures.

Vocal measure	Definition
Total vocalizations	Number of times the child produces a sound by vibrating their vocal folds during exhalation (i.e., eggressive phonation)
Communicative vocalizations	Number of identified vocalizations that occurred within a communication act Communication acts are defined as a behavior or set of behaviors that meet criteria for one of the following: (a) word(s) (spoken or signed), (b) nonword vocalization(s) with evidence of coordinated attention, (c) one of the 15 specific gestures (i.e., tapping with fingers/hand, clapping, reaching, proximal pointing, distal pointing, “shh” gesture, head nod or head shake, wave, shoulder shrug, pantomime-like actions and depictive gestures, moving object toward adult, upturned palm, giving object, showing object, and hand as tool) with evidence of coordinated attention to message/referent and communication partner, or (d) use of graphic symbol(s). It should be noted that participants have access to the Picture Exchange Communication System during the CCS scripted assessment protocol in this study.
Proportion of communicative vocalizations	Number of communicative vocalizations divided by the number of total vocalizations
Consonant inventory	Number of consonants out of 10 possible consonants (i.e., /m/, /n/, /b/ or /p/, /d/ or /t/, /g/ or /k/, /w/, /l/, “y,” /s/, and “sh”) the child used, within or outside a communication act Members of voiced–voiceless pairs (e.g., /b/ vs. /p/, /d/ vs. /t/, /g/ vs. /k/) are not distinguished due to reliability difficulties under the recording and coding conditions.
Diversity of key consonants used in communication acts	Number of consonants out of 10 possible consonants (i.e., /m/, /n/, /b/ or /p/, /d/ or /t/, /g/ or /k/, /w/, /l/, “y,” /s/, and “sh”) the child used *within communication acts* Members of voiced–voiceless pairs (e.g., /b/ vs. /p/, /d/ vs. /t/, /g/ vs. /k/) are not distinguished due to reliability difficulties under the recording and coding conditions.
Word approximations	Number of apparent attempts to produce spoken words Must meet (a) the pronunciation threshold, (b) be used in a semantically and pragmatically conventional manner, and (c) be analyzable content (e.g., not in singing).

*Note.* CCS = Communication Complexity Scale.

Trained research assistants and the first author coded the six vocal measures across the entire video recording of the CCS scripted administration protocol (mean time = 26.42 min, *SD* = 4.37 min). The coding manual is available from the first author upon request. Coders were required to achieve at least 80% small/large agreement (i.e., for the two values from the independent coders, the smaller value is divided by the larger value and multiplied by 100) for all measures with the first author prior to coding independently (Yoder et al., 2018). Recordings from 19% of the participants were coded by a second independent coder. The primary coder was blinded to which videos would be coded for reliability. We completed discrepancy discussions for all videos coded for reliability to prevent coder drift. ICCs with absolute agreement and participant and observer as random factors were calculated. The ICCs, which account for differences in unitizing and classifying the vocalizations and for variance among participants, ranged from .97 to .99 for the full sample length. See Table 3 for details. Mitchell (1979) interprets ICCs for .70 or greater as “very good.”

**Table 3.** Intraclass correlation coefficients by vocal measure for full sample.

Vocal measure	Intraclass correlation coefficient
TV	.98
CV	.98
PCV	.99
CI	.97
DKCC	.99
WA	.99

*Note.* TV = total vocalizations; CV = communicative vocalizations; PCV = proportion of communicative vocalizations; CI = consonant inventory; DKCC = diversity of key consonants used in communication acts (Wetherby et al., 2007; Woynaroski et al., 2017); WA = word approximations.

### Sample Lengths

After the complete video recordings of the CCS were coded for all six vocal measures, we calculated the values of each variable for nine different sample lengths. We selected lengths of 1, 3, 7, 10, and 20 min to align with prior work in the extant literature (Guo & Eisenberg, 2015; Heilmann et al., 2010). We used these lengths from the beginning of the sample and from the midpoint of each video recording. Time from midpoint was examined because a warm-up period is often recommended for communication samples. None of the videos exceeded 40 min. Therefore, we could not analyze 20-min samples from the midpoint. Thus, we analyzed 1-, 3-, 7-, and 10-min samples from the beginning and from the midpoint of the CCS video recording as well as the 20-min sample from the beginning and the full CCS sample. We calculated Cronbach's alpha as a measure of reliability between each of the shorter samples and the full CCS sample for the vocal measures. We used Cronbach's alpha because it is a measure of internal consistency, which describes the degree to which the test items measure the same concept (Cronbach, 1951).

## Results

Table 4 displays the means, standard deviations, and ranges for the vocal measures by sample length for those starting at the beginning of the CCS scripted administration protocol. Table 5 displays the analogous information for the samples that started at the midpoint of the CCS scripted administration protocol. As expected, all measures except the proportion of communicative vocalizations generally decrease as the length of the sample decreases. Because the proportion of communicative vocalizations is a proportion variable, it does not necessarily decrease for shorter sample lengths.

**Table 4.** Means (standard deviations) and ranges of vocal measures by sample length from the beginning of the Communication Complexity Scale (Brady et al., 2018).

Vocal measure	Entire sample		10 min		7 min		3 min		1 min	
	*M* (*SD*)	Range	*M* (*SD*)	Range	*M* (*SD*)	Range	*M* (*SD*)	Range	*M* (*SD*)	Range
TV	71 (45)	7–165	54 (36)	5–127	28 (21)	1–68	21 (15)	0–52	9.2 (7.0)	0–22
CV	13 (16)	0–79	8.9 (14)	0–71	5.1 (7.4)	0–39	3.3 (4.2)	0–21	1.3 (1.8)	0–6
PCV	0.19 (0.16)	0–0.60	0.16 (0.15)	0–0.64	0.18 (0.18)	0–0.57	0.17 (0.25)	0–0.56	0.16 (0.26)	0–0.44
CI	5.7 (3.3)	0–10	5.0 (3.4)	0–10	4.1 (3.1)	0–9	3.7 (2.9)	0–9	2.2 (2.3)	0–7
DKCC	3.1 (2.8)	0–8	2.4 (2.4)	0–8	1.9 (2.2)	0–8	1.5 (1.9)	0–8	0.81 (1.5)	0–6
WA	10 (18)	0–80	6.3 (14)	0–69	4.0 (8.0)	0–38	2.7 (5.2)	0–21	1.1 (2.2)	0–8

*Note.* TV = total vocalizations; CV = communicative vocalizations; PCV = proportion of communicative vocalizations; CI = consonant inventory; DKCC = diversity of key consonants used in communication acts (Wetherby et al., 2007; Woynaroski et al., 2017); WA = word approximations.

**Table 5.** means (standard deviations) and ranges of vocal measures by sample length from the midpoint of the Communication Complexity Scale (Brady et al., 2018).

Vocal measure	Entire sample		10 min		7 min		3 min		1 min		
	*M* (*SD*)	Range	*M* (*SD*)	Range	*M* (*SD*)	Range	*M* (*SD*)	Range	*M* (*SD*)	Range	
TV	71 (45)	7–165	26 (18)	0–61	19 (13)	0–52	7.6 (5.9)	0–25	2.3 (2.4)	0–10
CV	13 (16)	0–79	4.0 (4.3)	0–17	3.6 (4.3)	0–16	1.4 (2.1)	0–7	0.32 (0.79)	0–73
PCV	0.19 (0.16)	0–0.60	0.17 (0.14)	0–0.50	0.19 (0.17)	0–0.60	0.15 (0.21)	0–0.75	0.11 (0.22)	0–0.67
CI	5.7 (3.3)	0–10	3.9 (3.1)	0–10	3.6 (2.9)	0–10	2.0 (2.2)	0–9	1.1 (1.7)	0–7
DKCC	3.1 (2.8)	0–8	1.7 (2.3)	0–8	1.7 (2.0)	0–6	0.68 (1.2)	0–4	0.26 (0.82)	0–4
WA	10 (18)	0–80	2.8 (4.6)	0–18	3.0 (5.2)	0–24	1.1 (2.4)	0–9	0.23 (0.76)	0–4

*Note.* TV = total vocalizations; CV = communicative vocalizations; PCV = proportion of communicative vocalizations; CI = consonant inventory; DKCC = diversity of key consonants used in communication acts (Wetherby et al., 2007; Woynaroski et al., 2017); WA = word approximations.

For Research Question 1, Table 6 displays the results of the reliability analysis comparing the vocal measures from the shortened sample lengths with those from the full sample. Cronbach's alpha values range from 0 to 1. Values of at least .70 are generally considered acceptable reliability with those of at least .80 being very good (DeVellis, 1991; Nunnally, 1978). Values that exceed .80 are bolded in Table 6. As shown in Table 6, consonant inventory maintains Cronbach's alpha values of at least .80 for samples as short as 3 min. The proportion of communicative vocalizations also exceeds .80 for the 3-min samples from the midpoint, but not from the beginning. DKCC exceeds .80 with 7-min samples from the beginning or midpoint. Total vocalizations, communicative vocalizations, and word approximations exceed .80 with 10- or 20-min samples from the beginning, but not 10-min samples from the midpoint. As a group, the samples from the beginning of the CCS exhibited greater agreement with values from the full CCS samples than those from the midpoint of the CCS. Possible reasons for this finding are presented in the discussion section.

**Table 6.** Cronbach's alpha for vocal measures at different sample lengths.

Vocal measure	Length of sample								
	Minutes from beginning					Minutes from midpoint			
	20	10	7	3	1	10	7	3	1
TV	**.96**	**.82**	.69	.36	.12	.72	.62	.32	.12
CV	**.98**	**.84**	.62	.19	.08	.55	.53	.34	.09
PCV	**.99**	**.94**	**.86**	.55	.59	**.93**	**.93**	**.89**	.50
CI	**.99**	**.96**	**.95**	**.86**	.60	**.93**	**.92**	**.81**	.67
DKCC	**.98**	**.88**	**.81**	.61	.18	**.92**	**.90**	.62	.40
WA	**.97**	**.83**	.68	.26	.05	.57	.58	.32	.08

*Note.* Bolded values exceed .80. TV = total vocalizations; CV = communicative vocalizations; PCV = proportion of communicative vocalizations; CI = consonant inventory; DKCC = diversity of key consonants used in communication acts (Wetherby et al., 2007; Woynaroski et al., 2017); WA = word approximations.

For Research Question 2, Table 7 displays the Pearson product–moment correlations between the vocal measures from 10-min samples. All of the correlations exceed .40 and are statistically significant except for some of the correlations with the proportion of communicative vocalizations. Correlations among the vocal measures for other lengths are available in Supplemental Material S1. Except for the 1-min samples and the 3-min samples from the midpoint, total vocalizations, communicative vocalizations, consonant inventory, DKCC, and word approximations consistently significantly correlate with one another. The proportion of communicative vocalizations correlates with communicative vocalizations, DKCC, and word approximations for most sample lengths from the beginning and midpoint except for 1-min samples.

**Table 7.** Pearson product–moment correlations between vocal measures for 10-min samples.

Vocal measure	1.	2.	3.	4.	5.	6.
1. TV		.71**	−.15	.71**	.71**	.55**
2. CV	.55**		.44*	.41*	.73**	.82**
3. PCV	−.02	.61**		−.17	.23	.36
4. CI	.72**	.47**	.07		.72**	.55**
5. DKCC	.59**	.79**	.45*	.67**		.74**
6. WA	.57**	.93**	.48**	.53**	.85**	

*Note.* Values below the diagonal are from 10-min samples from the beginning of the sample. Values above the diagonal are from 10-min samples from the midpoint of the sample. Bolded values exceed .80. TV = total vocalizations; CV = communicative vocalizations; PCV = proportion of communicative vocalizations; CI = consonant inventory; DKCC = diversity of key consonants used in communication acts (Wetherby et al., 2007; Woynaroski et al., 2017); WA = word approximations.

* *p* < .05.

** *p* < .01.

These correlations highlight that the proportion of communicative vocalizations is more likely to correlate significantly with other variables that consider communicativeness than those that do not (i.e., total vocalizations and consonant inventory). Lower correlations may also be due to the proportion of communicative vocalizations being a proportion metric. Because proportion variables include a numerator and a denominator, errors from each combine for the measurement error of the proportion variables. Additional error is introduced for the proportion metric relative to the other count metrics (i.e., number of total vocalizations) due to low denominators for some participants (Yoder et al., 2018). More participants are likely to have low denominators for the shorter sample lengths, resulting in more measurement error for shorter samples. Increased measurement error can weaken correlations.

## Discussion

The current analysis tested the effect of the length of the CCS scripted administration protocol that was coded on the reliability and convergent validity of six theoretically and empirically supported vocal measures. Regarding reliability, consonant inventory showed very good reliability for 3-min samples (from the beginning or midpoint) with the full sample. No other vocal variable achieved sufficient reliability in this short of a sample length. Multiple factors may contribute to this relatively high reliability for short samples for consonant inventory. For example, the coder does not need to determine whether the vocalization was communicative and only needs to clearly hear the consonant once during the sample. Thus, the coding decisions may be easier for consonant inventory than for some of the other vocal measures. In addition, the exact number of instances of each consonant is not specified. If there is a single clear instance of a consonant in the sample, other less clear instances are not likely to influence the scoring. DKCC and proportion of communicative vocalizations exhibited very good reliability with the full sample with 7-min samples. Total vocalizations and communicative vocalizations achieved adequate reliability with 10-min samples from the beginning of the CCS scripted administration protocol (not from the midpoint). Thus, coding a 10-min portion of the CCS scripted administration protocol may be reasonable for each of the tested vocal measures. The 10-min sample would provide flexibility in which variable(s) could be coded. However, if a clinician or researcher is interested in a specific vocal variable that showed high reliability with shorter samples (e.g., consonant inventory), shorter samples would suffice.

Overall greater agreement was observed between samples from the beginning of the CCS with the full CCS samples, rather than those from the midpoint with the full CCS samples. Additional investigations are needed to test whether the difference between samples from the beginning versus the midpoint replicates and to identify the specific reason(s) for this difference. Some potential reasons relate to the activities and interaction between the examiner and child across the session. For example, the activities are generally presented in the same order during the CCS, and some activities may be easier to score than others.

Regarding convergent validity, the communication sample length had little impact on the correlations between the vocal measures until the samples were only a minute in length. These correlations provide strong evidence of convergent validity for all of the vocal measures. Although the proportion of communicative vocalizations correlated with some selected vocal measures more consistently than other vocal measures, it tended to correlate more highly with other vocal variables that also consider communicativeness. Overall, the results extend prior findings of evidence of convergent validity for vocal measures in children with ASD obtained from samples as short as 3 min or even 1 min and to the context of the CCS scripted assessment protocol (McDaniel et al., 2018, 2020b; Wetherby et al., 2007; Yoder et al., 2015).

Combined, the reliability and convergent validity analyses support the use of the CCS scripted administration protocol as a sampling context for assessing vocal development in children with ASD. The results provide evidence for coding a 10-min sample of vocalizations produced during the CCS. Coding shorter samples is feasible in clinical and research settings with sufficient training. For the current project, undergraduate students pursuing degrees in speech-language-hearing were trained to code the vocal variables reliably. In general, learning to code vocal variables that consider communicativeness required more time than those that do not consider communicativeness because coding the communicative vocal variables required judgments of intentionality in addition to phonemic discriminations.

### Limitations and Future Directions

Replication of the findings, as well as expansion of them to more diverse samples of children, such as children with disabilities other than ASD, is needed. Expanding the sample's size and diversity will permit investigation of child characteristics that influence the reliability and validity of the vocal measures for capturing and predicting progress. As shown in prior work, children at older ages with relatively higher skills required shorter sessions to achieve adequate stability of communication variables than younger children (McDaniel et al., 2021; Sandbank & Yoder, 2014). Additional empirical evaluation is needed to determine the degree to which age and skill level influence the reliability and validity of vocal measures coded during the CCS.

This study evaluated reliability and convergent validity between vocal measures. Future work to evaluate the predictive validity, divergent validity, and sensitivity to change would provide important information for selecting a subset of vocal measures that could be used to measure progress in vocalizations or to predict gains in speech. For example, one vocal measure may require 7-min samples for sufficient reliability, and another may require 3-min samples for sufficient reliability. However, if the first accounts for more variance in future language outcomes, the additional time spent coding the 7-min samples may be warranted. Evidence on the sensitivity to change would provide guidance on how to interpret change over time for making intervention decisions. For example, is a particular child exhibiting more or less growth than expected for a child who will progress past minimal verbal skills? The amount of training time for different variables also warrants further investigation. If two vocal measures exhibit similar validity but one requires much less training time than the other, using the vocal measure that requires less training time would be beneficial.

Future research is needed to investigate whether results are specific to the CCS context or whether they could be extended to other sampling contexts. For example, the CCS protocol tasks are presented in a specified order, and it may be that certain tasks are more likely to evoke vocal behaviors than others. Identifying high-yield activities could reduce or simplify further the task of coding vocal measures from the CCS scripted administration protocol. Additionally, the CCS provides a structured context for the communication sample. Vocal responding during the CCS may differ from less structured contexts in homes and classrooms. Future research is needed to determine the representativeness of the vocalizations derived from the CCS compared to other contexts.

## Conclusions

In summary, results of this study support coding short 10-min segments from the CCS scripted administration protocol to evaluate the vocal development skills of children with ASD who have not yet achieved phrase speech. All of the tested vocal measures exhibited very good reliability with 10-min samples. Consonant inventory exhibited the highest reliability with the shortest samples. Sample length showed limited influence on convergent validity. The findings provide an empirically supported method for efficiently extracting additional information from video-recorded interactions such as the CCS scripted administration protocol in clinical and research settings for children with ASD who use few or no words and have limited assessment options (Tager-Flusberg & Kasari, 2013).

## Data Availability Statement

The data sets generated during and/or analyzed during the current study are available from the corresponding author on reasonable request.

## Supplementary Material

10.1044/2022_JSLHR-22-00010SMS1 Supplemental Material S1 Pearson product moment correlations between vocal measures by sample length.
