# Supplementary material for: The Influence of Communication Sample Length on Reliability and Convergent Validity of Vocal Measures Derived From the Communication Complexity Scale
Source: J Speech Lang Hear Res. 2022 Sep 12;65(10):3881–9. doi: 10.1044/2022_JSLHR-22-00010 (PMC9927626; doi:10.1044/2022_JSLHR-22-00010)
Supplement: Supplemental Material S1 [file JSLHR-65-3881-s001.pdf]

Supplemental Material S1. Pearson product moment correlations between vocal measures by sample length.

Pearson Product Moment Correlations Between Vocal Measures for 1-Minute Samples

Vocal measure	1.	2.	3.	4.	5.	6.
1. TV		.38*	.19	.86**	.34	.35
2. CV	.39*		.93**	.35	.85**	.71**
3. PCV	–.09	.77**		.17	.68**	.44*
4. CI	.42*	.26	–.06		.46**	.49**
5. DKCC	.23	.66**	.34	.42*		.87**
6. WA	.17	.05	–.09	.62**	.31	

Note. Values below the diagonal are from 1-minute samples from the beginning of the sample. Values above the diagonal are from 1-minute samples from the midpoint of the sample. CI = consonant inventory; CV = communicative vocalizations; DKCC = diversity of key consonants used in communication acts (Wetherby et al., 2007; Woynaroski et al., 2017); PCV = proportion of communicative vocalizations; TV = total vocalizations; WA = word approximations.
*$p < .05$. **$p < .01$.

Pearson Product Moment Correlations Between Vocal Measures for 3-Minute Samples

Vocal measure	1.	2.	3.	4.	5.	6.
1. TV		.47**	.25	.78**	.42*	.33
2. CV	.47**		.94**	.37*	.87**	.87**
3. PCV	–.21	.56**		.21	.77**	.82**
4. CI	.76**	.72**	.20		.44*	.33
5. DKCC	.42*	.79**	.43*	.74**		.82**
6. WA	.56**	.84**	.28	.65**	.68**	

Note. Values below the diagonal are from 3-minute samples from the beginning of the sample. Values above the diagonal are from 3-minute samples from the midpoint of the sample. CI = consonant inventory; CV = communicative vocalizations; DKCC = diversity of key consonants used in communication acts (Wetherby et al., 2007; Woynaroski et al., 2017); PCV = proportion of communicative vocalizations; TV = total vocalizations; WA = word approximations.
*$p < .05$. **$p < .01$.

Pearson Product Moment Correlations Between Vocal Measures for 7-Minute Samples

Vocal measure	1.	2.	3.	4.	5.	6.
1. TV		.58**	.02	.76**	.69**	.46*
2. CV	.47**		.70**	.49**	.75**	.88**
3. PCV	–.13	.60**		.09	.44*	.58**
4. CI	.70**	.55**	.11		.80**	.53**
5. DKCC	.52**	.84**	.49**	.65**		.71**
6. WA	.47**	.91**	.44*	.58**	.90**	

Note. Values below the diagonal are from 7-minute samples from the beginning of the sample. Values above the diagonal are from 7-minute samples from the midpoint of the sample. CI = consonant inventory; CV = communicative vocalizations; DKCC = diversity of key consonants used in communication acts (Wetherby et al., 2007; Woynaroski et al., 2017); PCV = proportion of communicative vocalizations; TV = total vocalizations; WA = word approximations.
*$p < .05$. **$p < .01$.

Pearson Product Moment Correlations Between Vocal Measures for 10-Minute Samples

Vocal measure	1.	2.	3.	4.	5.	6.
1. TV		.71**	–.15	.71**	.71**	.55**
2. CV	.55**		.44*	.41*	.73**	.82**
3. PCV	–.02	.61**		–.17	.23	.36
4. CI	.72**	.47**	.07		.72**	.55**
5. DKCC	.59**	.79**	.45*	.67**		.74**
6. WA	.57**	.93**	.48**	.53**	.85**	

Note. Values below the diagonal are from 10-minute samples from the beginning of the sample. Values above the diagonal are from 10-minute samples from the midpoint of the sample. CI = consonant inventory; CV = communicative vocalizations; DKCC = diversity of key consonants used in communication acts (Wetherby et al., 2007; Woynaroski et al., 2017); PCV = proportion of communicative vocalizations; TV = total vocalizations; WA = word approximations.
**p* < .05. ***p* < .01.

Pearson Product Moment Correlations Between Vocal Measures for 20-Minute Samples from the Beginning of the Sample

Vocal measure	1.	2.	3.	4.	5.	6.
1. TV						
2. CV	.54**					
3. PCV	.07	.73**				
4. CI	.70**	.38*	.09			
5. DKCC	.68**	.62**	.39*	.77**		
6. WA	.51**	.94**	.64**	.41*	.67**	

Note. CI = consonant inventory; CV = communicative vocalizations; DKCC = diversity of key consonants used in communication acts (Wetherby et al., 2007; Woynaroski et al., 2017); PCV = proportion of communicative vocalizations; TV = total vocalizations; WA = word approximations.
**p* < .05. ***p* < .01.

Pearson Product Moment Correlations Between Vocal Measures for the Full Communication Complexity Scale Sample

Vocal measure	1.	2.	3.	4.	5.	6.
1. TV						
2. CV	.56**					
3. PCV	.00	.71**				
4. CI	.64**	.43*	.10			
5. DKCC	.62**	.66**	.43*	.79**		
6. WA	.50**	.91**	.59**	.51**	.69**	

Note. CI = consonant inventory; CV = communicative vocalizations; DKCC = diversity of key consonants used in communication acts (Wetherby et al., 2007; Woynaroski et al., 2017); PCV = proportion of communicative vocalizations; TV = total vocalizations; WA = word approximations.
**p* < .05. ***p* < .01.

References

Wetherby, A. M., Watt, N., Morgan, L., & Shumway, S. (2007). Social communication profiles of children with autism spectrum disorders late in the second year of life. *Journal of Autism and Developmental Disorders, 37*(5), 960–975. https://doi.org/10.1007/s10803-006-0237-4

Woynaroski, T., Oller, D. K., Keceli-Kaysili, B., Xu, D., Richards, J. A., Gilkerson, J., Gray, S., & Yoder, P. (2017). The stability and validity of automated vocal analysis in preverbal preschoolers with autism spectrum disorder. *Autism Research, 10*(3), 508–519. https://doi.org/10.1002/aur.1667
